# Uraemic toxins impair skeletal muscle regeneration by inhibiting myoblast proliferation, reducing myogenic differentiation, and promoting muscular fibrosis

**DOI:** 10.1038/s41598-020-79186-1

**Published:** 2021-01-12

**Authors:** Elena Alcalde-Estévez, Patricia Sosa, Ana Asenjo-Bueno, Patricia Plaza, Gemma Olmos, Manuel Naves-Díaz, Diego Rodríguez-Puyol, Susana López-Ongil, María P. Ruiz-Torres

**Affiliations:** 1 Departamento de Biología de Sistemas, Facultad de Medicina Y Ciencias de La Salud, Universidad de Alcalá, 28871 Alcalá de Henares, Madrid, Spain; 2 Unidad de Investigación de La Fundación Para La Investigación Biomédica del Hospital Universitario Príncipe de Asturias, Alcalá de Henares, Madrid, Spain; 3 Instituto Reina Sofía de Investigación Nefrológica, IRSIN, Madrid, Spain; 4 Area 3-Fisiología y Fisiopatología Renal Y Vascular del IRYCIS, Madrid, Spain; 5 Unidad de Gestión Clínica de Metabolismo Óseo. Hospital Universitario Central de Asturias, ISPA, Oviedo, Spain; 6 Departamento de Medicina Y Especialidades Médicas, Universidad de Alcalá Y Servicio de Nefrología del Hospital Universitario Príncipe de Asturias, Alcalá de Henares, Madrid, Spain

**Keywords:** Kidney diseases, Cell biology

## Abstract

Uraemic toxins increase in serum parallel to a decline in the glomerular filtration rate and the development of sarcopenia in patients with chronic kidney disease (CKD). This study analyses the role of uraemic toxins in sarcopenia at different stages of CKD, evaluating changes in the muscular regeneration process. Cultured C_2_C_12_ cells were incubated with a combination of indoxyl sulphate and p-cresol at high doses (100 µg/mL) or low doses (25 µg/mL and 10 µg/mL) resembling late or early CKD stages, respectively. Cell proliferation (analysed by scratch assays and flow cytometry) was inhibited only by high doses of uraemic toxins, which inactivated the cdc2-cyclin B complex, inhibiting mitosis and inducing apoptosis (analysed by annexin V staining). By contrast, low doses of uraemic toxins did not affect proliferation, but reduced myogenic differentiation, primed with 2% horse serum, by inhibiting myogenin expression and promoting fibro-adipogenic differentiation. Finally, to assess the in vivo relevance of these results, studies were performed in gastrocnemii from uraemic rats, which showed higher collagen expression and lower myosin heavy chain expression than those from healthy rats. In conclusion, uraemic toxins impair the skeletal muscular regeneration process, even at low concentrations, suggesting that sarcopenia can progress from the early stages of CKD.

## Introduction

Sarcopenia is an aging-related condition, characterised by progressive loss of muscle mass and strength that results in impaired physical performance that has a dramatic impact on health. Patients affected by chronic diseases, such as hypertension, diabetes, and chronic kidney disease (CKD), have a high prevalence of sarcopenia^1^. Patients with CKD display cachexia and sarcopenia that contribute to frailty and morbidity^2^. Cachexia is considered a metabolic syndrome associated with underlying illness and characterised by a loss of muscle, with or without loss of fat, and with unintended weight loss. It is frequently related to an inflammatory process that increases catabolic metabolism. Sarcopenia is considered to be an important feature in the pathophysiology of the cachexia phenotype^3,4^, but the mechanisms involved in its appearance are only partially understood. Changes in muscles related to sarcopenia include type II fibre atrophy, fibre size reduction, problems with the connective tissue and fat accumulation between the fibres, failure of mitochondrial metabolism resulting in decreasing oxidative capacity, increased protein catabolism and inflammation, and the weakening of the muscular regeneration process^5–7^.

The number and function of satellite cells, the myogenic stem cells, is reduced in sarcopenic muscle, which can compromise the regenerative capacity of muscles^7^. Satellite cells are quiescent and are activated after muscle damage or growth stimulus. Activated satellite cells, called myoblasts, begin to proliferate. They can generate the necessary myogenic progenitors to form new muscle fibres or they can return to a quiescent state to maintain the set of satellite cells. The cell fate decision is determined by intrinsic or extrinsic factors, which are present in the microenvironment near the satellite cell niche^7^.

It is estimated that about 37% of dialysis patients display symptoms of sarcopenia^8^ and this has been correlated with an increased risk of mortality^8,9^. Causes of the development of sarcopenia in CKD patients are related to protein energy waste, low physical activity^10^, and some inflammatory cytokines^11^, which contribute to muscle wasting and the loss of muscular strength. Also, an important role for uraemia in sarcopenia has recently been described, as the progression of sarcopenia correlates with the glomerular filtration rate reduction in patients with CKD^12^. Uraemia is a toxic condition caused by the accumulation of urea and other nitrogenous waste compounds (uraemic toxins, UT) in the blood^13^ which are not efficiently eliminated by the kidneys due to a decline in the glomerular filtration rate. The term uraemic sarcopenia refers to the marked effect that the rise in UT has on the development of skeletal muscle abnormalities^14^. UT are categorised as free water-soluble or protein-bound molecules and can modify biological functions^15^. Among UT, indoxyl sulphate (IS) and the conjugates of p-cresol (PC) are protein-binding low-molecular-weight compounds produced by gut microbiota. IS is a metabolite derived from tryptophan and PC from the amino acids phenylalanine and tyrosine^16^. Both of them are hard to eliminate by classical dialysis methods because they are strongly bonded to serum proteins^17^. The toxicity of UT in many organs has been studied. However, at present, the specific contribution of UT to the development of sarcopenia is not well understood. In this sense, high levels of IS inhibit myoblast proliferation and myogenic differentiation^18,19^, promote metabolic alterations^20^, induce atrophy of myotubes through the production of reactive oxygen species^21^, and promote mitochondrial dysfunction^22^. The role of PC and its conjugates in sarcopenia, by contrast, has been less explored.

Here, we hypothesise that sarcopenia is initiated during the first steps of CKD by the effect of UT. We evaluate the effects of UT on the muscular regeneration process, and provide further insight into the intracellular mechanisms involved. Two different stages of CKD, were modelled using two different doses of IS and PC.

## Results

### High doses of UT reduced the myoblast proliferation capacity by inhibiting entry in phase M and by inducing apoptosis

**Figure 1:**
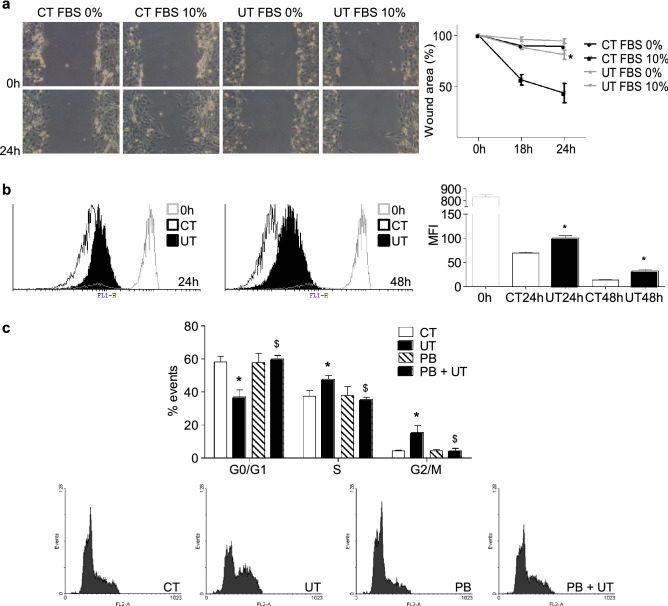
High doses of UT reduced the proliferation capacity of cultured murine myoblasts, arresting the cell cycle between the phases S and G2/M. C_2_C_12_ cells were treated with a mixture of 100 µg/mL IS and 100 µg/mL PC (UT) or vehicle (CT) for 48 h. (**a**) Scratch wounding was performed on the C_2_C_12_ monolayer and wound closure was monitored over the next 24 h in the presence or absence of 10% FBS. Photographs of wounds were captured at 0, 18, and 24 h post-wounding to determine the degree of wound closure. Representative experiment shows only the 0 and 24 h post-wounding photographs. Graph represents the percentage of control (time 0 h) wound area at different times post-wounding from five experiments. *p < 0.05 between CT FBS 10% and UT FBS 10%. (**b**) Cell proliferation was monitored using CellTrace CFSE solution by flow cytometry during 24 and 48 h. A representative experiment is shown. The bar graph represents the mean fluorescence intensity (MFI). The results are mean ± SEM from five experiments. *p < 0.05 versus CT at the same time. (**c**) C_2_C_12_ cells were treated for 48 h in culture medium with 10% FBS in the presence or absence of 0.25 mM probenecid (PB). Propidium iodide incorporation was assessed by flow cytometry. A representative experiment is shown. The bar graph represents the mean ± SEM from four experiments. *p < 0.05 versus CT. ^$^*p* < 0.05 versus UT.

C_2_C_12_ cells were treated with a mixture of 100 µg/mL IS and 100 µg/mL PC (high doses of UT), for 24 and 48 h. These doses resemble the UT serum levels found in the advanced phases of CKD. The effect of high doses of UT on the proliferation capacity of myoblasts was analysed using a scratch wound healing assay. Myoblasts were treated with high doses of UT or vehicle for 24 or 48 h. Next, UT were removed, and cells were stimulated to proliferate with standard culture medium complemented with 10% foetal bovine serum (FBS) for 24 h. A significant delay in wound closure was observed in cells treated with UT compared to control cells (Fig. 1a). Cellular proliferation was also evaluated using the CellTrace CFSE solution during UT treatment by flow cytometry. The results indicate that cells treated with UT suffered a significant decrease in the proliferation rate since the probe remained higher than in the vehicle-treated cells (Fig. 1b). The effect of high doses of UT on the cell cycle was evaluated via the incorporation of propidium iodide by flow cytometry. The results showed that proliferating cells treated with UT suffered a dramatic cell cycle arrest between the phases S and G2/M (Fig. 1c). To analyse the specificity of the UT effect, experiments were performed in the presence or absence of 0.25 mM probenecid. The results demonstrated that probenecid prevented the effect of UT (Fig. 1c). Only results obtained after 48 h of UT treatment are shown, but similar results were found after 24 h of UT treatment.

**Figure 2:**
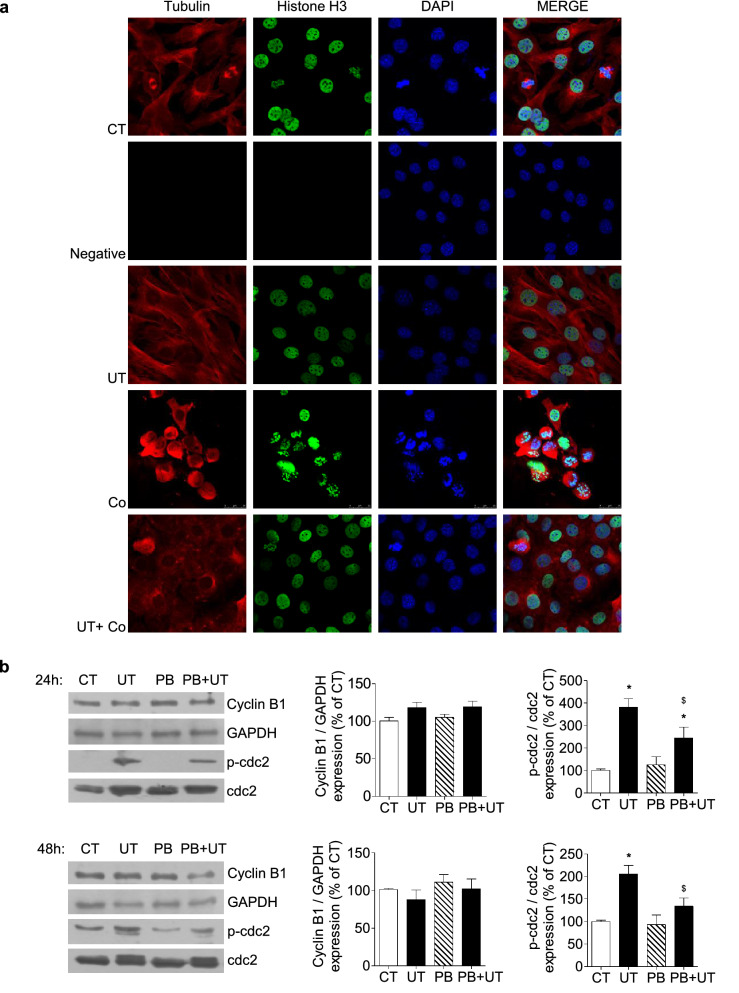
High doses of UT prevented entry into phase M promoting cdc2 phosphorylation and inactivating the Cyclin B/cdc2 complex. (**a**) C_2_C_12_ cells were treated with a mixture of 100 µg/mL IS and 100 µg/mL PC (UT) for 24 h in the presence or absence of 0.1 mg/mL colcemid (Co). Tubulin (red) and Histone H3 (green) expression and DAPI (blue) were analysed by immunofluorescence by using a confocal microscope. A representative experiment with negative staining control is shown. (**b**) C_2_C_12_ cells were incubated with a mixture of 100 µg/mL IS and 100 µg/mL PC (UT) or vehicle (CT) for 24 or 48 h in the presence or absence of 0.25 mM probenecid (PB). Cyclin B and phospho-cdc2 and cdc2 expression were analysed by western blotting. Representative blots are shown. Full-length blots are presented in Supplementary Figure S3. Bar graphs represent the densitometric analysis of the bands. Results are expressed as a percentage of control and are the mean ± SEM from five experiments. *p < 0.05 versus CT. ^$^*p* < 0.05 versus UT.

To analyse the mechanisms involved in the cell cycle arrest, chromosome condensation in the presence or absence of colcemid (0.1 µg/mL) was analysed in cells treated with or without UT for 24 h by immunostaining and confocal microscopy. It was found that in the presence of colcemid, vehicle-treated cells condensed their chromosomes, measured as H3 expression, as expected, whereas UT-treated cells did not, suggesting that UT stop the cell cycle at any point before the entry of cells into the mitotic phase (Fig. 2a). Additional experiments were performed with a mixture of 100 µg/mL IS plus an equivalent dose of p-cresyl sulphate (PCS) (226 µg/mL) instead of PC, to analyse whether the mixtures IS plus PC and IS plus PCS have similar effects on myoblast proliferation and chromosome condensation. Results presented in Supplementary Figure S1 indicate that cells treated with IS plus PCS did not condense their chromosomes and have reduced proliferation rate in the same way as cells treated with IS plus PC.

Next, the expression of cyclin B1 and phosphorylation of cyclin-dependent kinase 1 (cdc2) were analysed to evaluate whether UT induce changes in the G2/M checkpoint. No significant changes were found in cyclin B1 expression at either 24 or 48 h (Fig. 2b). In contrast, there was strong phosphorylation of cdc2 in the presence of UT, indicating that cdc2 and the complex cdc2-cyclin B were inactive. Again, the effect of UT was abolished in the presence of probenecid (Fig. 2b). This result could explain why cells did not enter the mitosis phase under UT exposition.

**Figure 3:**
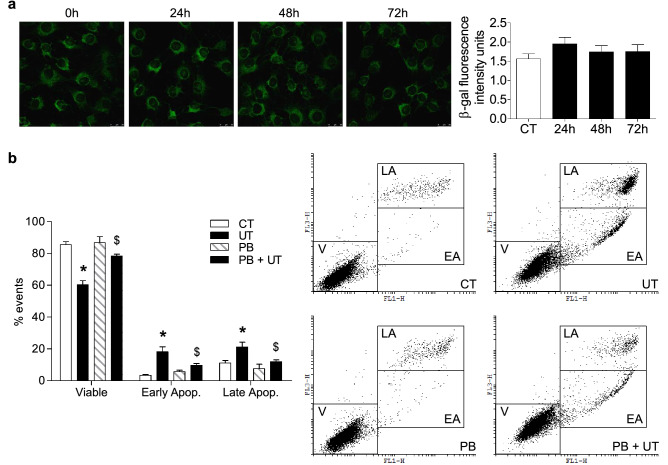
High doses of UT did not promote cell senescence, but they induced apoptosis in cultured myoblasts. (**a**) C_2_C_12_ cells were treated with a mixture of 100 µg/mL IS and 100 µg/mL PC (UT) for 24, 48, or 72 h. Senescence-associated β-galactosidase activity was analysed using confocal microscopy using the fluorogenic substrate C_12_FDG. A representative experiment is shown. The bar graph represents mean ± SEM from ten experiments. (**b**) C_2_C_12_ cells were treated with a mixture of 100 µg/mL IS and 100 µg/mL PC (UT) for 48 h in the presence or absence of 0.25 mM probenecid (PB). Apoptosis was analysed using the Annexin V test by flow cytometry. A representative experiment is shown. The bar graph represents the mean ± SEM from ten experiments. *p < 0.05 versus CT. ^$^*p* < 0.05 versus UT.

Next, we evaluated whether high doses of UT induced apoptosis or senescence in C_2_C_12_ cells. Senescence-associated β-galactosidase (SA-β-gal) activity was evaluated 24, 48, and 72 h after UT addition. No changes were found in SA-β-gal activity at any time, indicating that they did not induce cellular senescence (Fig. 3a). In contrast, UT induced the death of proliferating C_2_C_12_ cells by apoptosis, assessed by flow cytometry by the annexin V test after 48 h of UT treatment (Fig. 3b). This effect was abolished by probenecid (Fig. 3b).

### Low doses of UT induced neither changes in the cell cycle nor cellular senescence but diminished myogenic differentiation of cultured myoblasts

**Figure 4:**
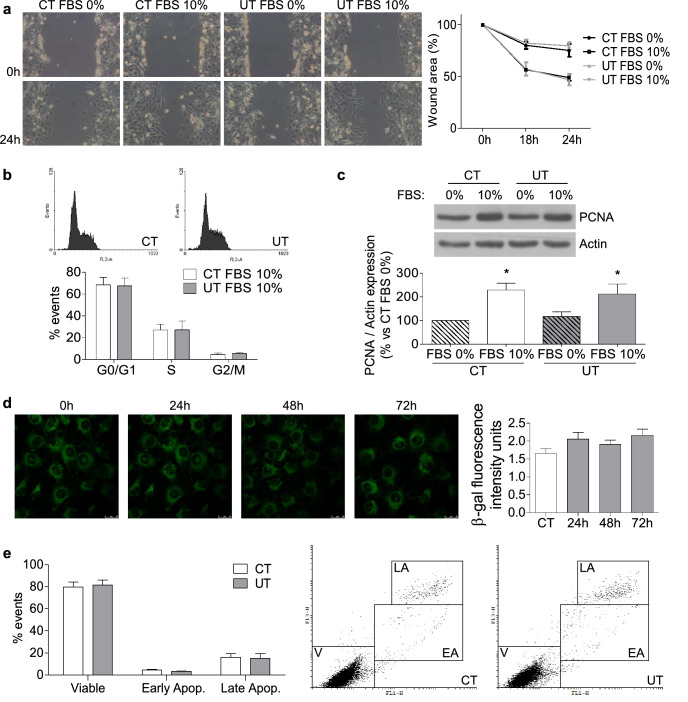
Low doses of UT did not change either the cell cycle or cellular senescence in murine cultured myoblasts. C_2_C_12_ cells were treated with a mixture of 25 µg/mL IS and 10 µg/mL PC (UT) or vehicle (CT) for 48 h. (**a**) Scratch wounding was made in the confluent monolayer and wound closure was monitored over the next 24 h with or without 10% FBS. Wounds were photographed at 0, 18, and 24 h after wounding to measure the degree of wound closure. A representative experiment shows only the photographs taken at 0 and 24 h after wounding. The graph represents the percentage of wound closure with respect to time 0 h. The results showed are mean ± SEM from five experiments. (**b**) C_2_C_12_ cells were treated for 48 h in culture medium with 10% FBS. Propidium iodide incorporation was assessed by flow cytometry. A typical experiment is shown. Results are the mean ± SEM from four experiments. (**c**) C_2_C_12_ cells were treated for 48 h with or without 10% FBS. PCNA expression was analysed by western blotting. A typical blot is shown. Full-length blot is presented in Supplementary Figure S3. The bar graph shows the densitometric analysis of the bands. The results, expressed as a percentage of control, are the mean ± SEM from seven different experiments. *p < 0.05 versus CT FBS 0%. (**d**) C_2_C_12_ cells were treated for 24, 48, or 72 h. Senescence-associated β-galactosidase activity was measured by confocal microscopy with the fluorogenic substrate C_12_FDG. A typical experiment is shown. Results showed in the bar graph represent mean ± SEM from ten experiments. (**e**) C_2_C_12_ cells were treated for 48 h. Apoptosis was analysed using the Annexin V test via flow cytometry. A typical experiment is shown. Results are the mean ± SEM from six experiments.

To analyse whether lower doses of UT have any effect on myoblast biology, C_2_C_12_ cells were treated with a mixture of 25 µg/mL IS and 10 µg/mL PC (low doses of UT), for 24 or 48 h. These doses resemble the UT concentration found in the early stages of CKD. To analyse whether low doses of UT reduce the proliferation of myoblasts, different approaches were utilised. First, a scratch wound healing assay was performed on a monolayer of C_2_C_12_ cells treated or not with low doses of UT for 24 or 48 h, using 10% FBS to stimulate the wound closure over 24 h. It was observed that there were no changes in the dynamics of wound closure in cells treated with UT compared with control cells (Fig. 4a). As to whether the UT were affecting the cell cycle, the propidium iodide incorporation was analysed by flow cytometry in cells with or without low doses of UT, using 10% FBS as the proliferative stimulus. The results showed that no changes were induced by UT at any time in the cell cycle (Fig. 4b). In addition, proliferating cell nuclear antigen (PCNA) expression was analysed in the presence or absence of 10% FBS with low doses of UT. Treatment with UT did not modify the expression of PCNA compared with control cells (Fig. 4c). Next, we analysed whether low doses of UT induce cellular senescence or apoptosis. SA-β-gal activity was evaluated 24, 48, and 72 h after UT addition. No changes were found in the SA-β-gal activity at any time point analysed, indicating that UT did not induce cellular senescence (Fig. 4d). Apoptosis was measured by annexin V staining and flow cytometry, and the results indicated no changes in the percentage of apoptotic cells (Fig. 4e). In all cases, the results were similar after 24 h of treatment, but only results obtained at 48 h are shown. All together, these results indicate that UT did not reduce the proliferative capacity of cultured myoblasts or induce cellular senescence or apoptosis.

**Figure 5:**
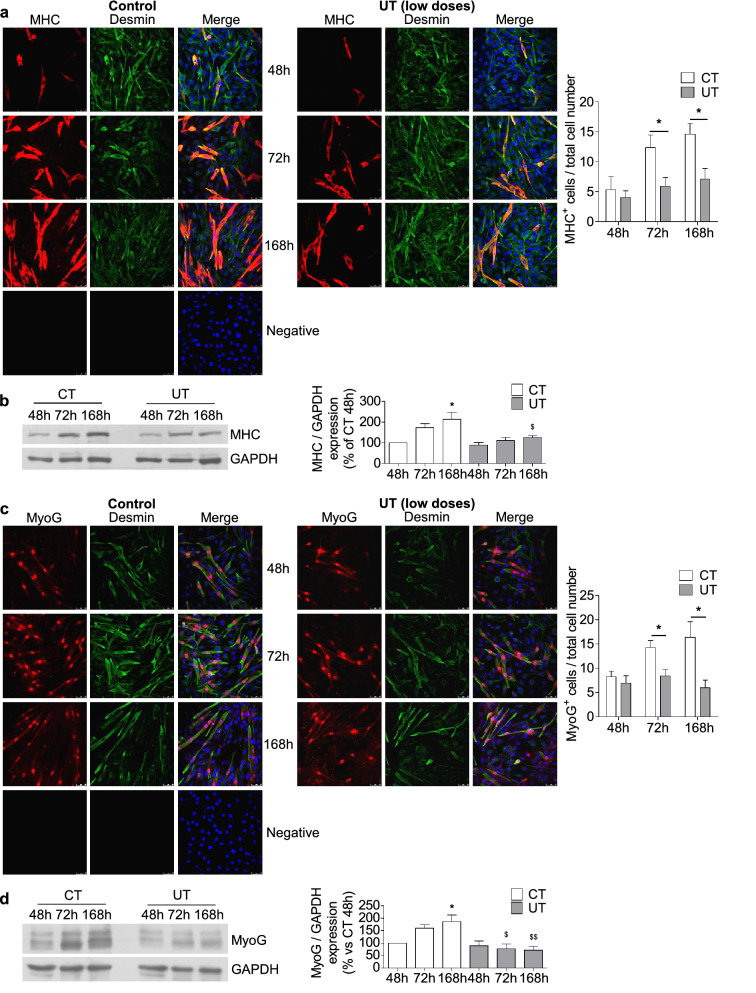
Low doses of UT impaired the myogenic differentiation of murine cultured myoblasts by diminishing MyoG expression. C_2_C_12_ cells grew with 2% horse serum for 48, 72, and 168 h to allow myogenic differentiation in the presence of a mixture of 25 µg/mL IS and 10 µg/mL PC (UT) or vehicle (CT). (**a**) Myosin heavy chain (MHC, red) and desmin (green) were evaluated through immunofluorescence using a confocal microscope. A typical experiment is shown in the left panel. Negative staining control is shown. Bar graph shows the mean ± SEM of MHC-positive cells, from nine experiments (right panel). *p < 0.05 versus CT at the same time. (**b**) MHC expression was measured by western blotting. A typical blot is shown. Full-length blot is presented in Supplementary Figure S3. The densitometric analysis of the bands is represented in bar graph. Results are the percentage respect to control and are the mean ± SEM from ten experiments. *p < 0.05 versus CT 48 h. ^$^*p* < 0.05 versus CT 168 h. (**c**) Myogenin (MyoG, red) and desmin (green) were determined by immunofluorescence and confocal microscopy. A typical experiment is shown in the left panel. Negative staining control is shown. The bar graph shows the mean ± SEM of MyoG-positive cells, from 10 experiments (right panel). *p < 0.05 versus CT at the same time. (**d**) MyoG expression was quantified by western blotting. A typical blot is shown. Full-length blot is presented in Supplementary Figure S3. The bar graph shows the densitometric analysis of the bands. Results are the percentage of control 48 h and are the mean ± SEM from six experiments. *p < 0.05 versus CT 48 h. ^$^*p* < 0.05 versus CT 72 h. ^$$^*p* < 0.05 versus CT 168 h.

To analyse the effects of low doses of UT on myoblasts differentiation, cultured C_2_C_12_ cells were maintained for seven days with culture medium supplemented with 2% horse serum to allow myogenic differentiation, with or without low doses of UT. Myotube formation was evaluated, at 48, 72, and 168 h after UT addition, as myosin heavy chain (MHC)-positive cells using immunofluorescent staining and confocal microscopy. C_2_C_12_ cells treated with UT showed a diminished myotube formation between 72 and 168 h with respect to cells treated with the vehicle for the same number of hours (Fig. 5a). The expression of MHC increased sequentially in the control cells in contrast with cells subjected to UT treatment (Fig. 5b), in which the increase was lower. Then, we analysed the expression of myogenin (MyoG), which regulates the myogenic differentiation, by western blotting and immunofluorescent staining. The results showed that MyoG expression, analysed through immunocytochemistry, increased in control cells after 72 h of culture coinciding with the initiation of myotube formation. By contrast, it was lower in UT-treated cells, suggesting that UT inhibited the expression of this factor (Fig. 5c). MyoG expression was also analysed by western blotting, and the same results were found (Fig. 5d).

### Low doses of UT promote fibrogenic and adipogenic differentiation in murine cultured myoblasts

**Figure 6:**
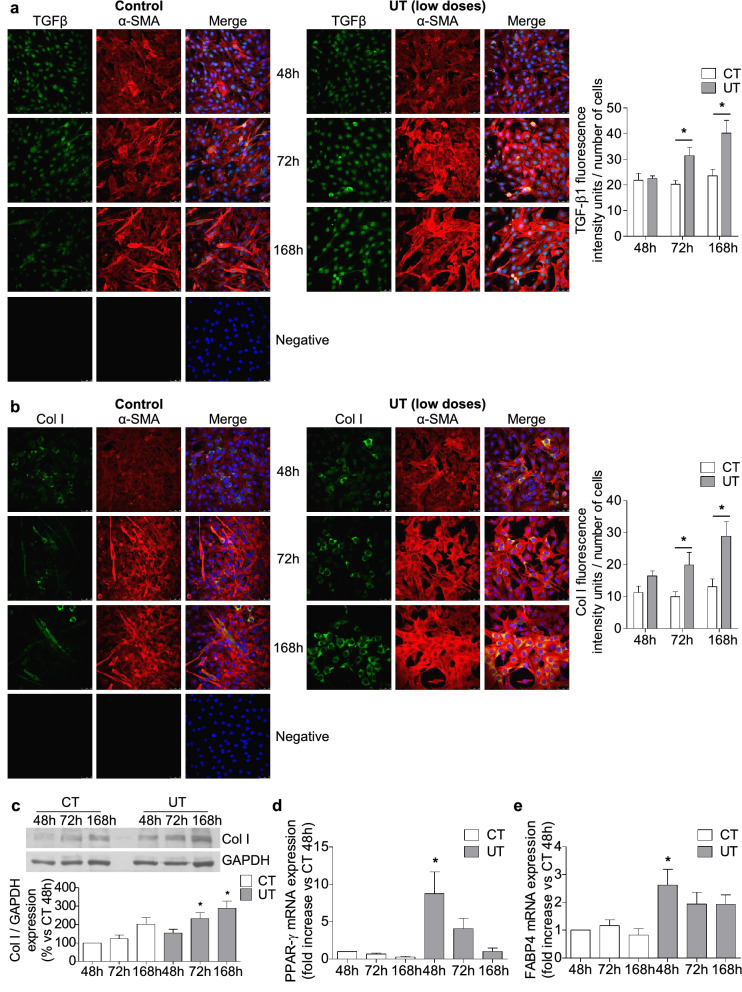
Low doses of UT promoted fibrogenic and adipogenic differentiation in murine cultured myoblast. C_2_C_12_ grew with 2% horse serum for 48, 72, and 168 h to allow myogenic differentiation in the presence of a mixture of 25 µg/mL IS and 10 µg/mL PC (UT) or vehicle (CT). (**a**) TGF-β1 (green) and α-Smooth muscle actin (α-SMA, red) expression were analysed by immunofluorescence using a confocal microscope. A typical experiment is shown in the left panel. Negative staining control is shown. The bar graph shows the analysis of the intensity of green fluorescence (TGF-β1) corrected by the number of cells. Results, expressed as arbitrary fluorescence intensity units, are the mean ± SEM from ten different experiments. *p < 0.05 versus CT at the same time. (**b**) Collagen I expression (Col I, green) and α-SMA (red) were analysed by immunofluorescence using a confocal microscope. A typical experiment is shown in the left panel. Negative staining control is shown. The bar graph represents the densitometric analysis of the green fluorescence (Col I) corrected by the number of cells. Results are expressed as arbitrary fluorescence intensity units and are the mean ± SEM from ten different experiments. *p < 0.05 versus CT at the same time. (**c**) Col I protein expression was quantified by western blotting. A typical blot is shown. Full-length blot is presented in Supplementary Figure S3. The bar graph shows the densitometric analysis of the bands. Results are percentage of control 48 h and are the mean ± SEM from nine experiments. *p < 0.05 versus CT 48 h. (**d**) PPAR-γ mRNA expression was analysed by RT-PCR. Results are mean ± SEM from four experiments. *p < 0.05 versus CT 48 h. (**e**) FABP4 mRNA expression was analysed by RT-PCR. Results are mean ± SEM from four experiments. *p < 0.05 versus CT 48 h.

To analyse whether low doses of UT are changing the cell fate and inducing fibrogenic differentiation, C_2_C_12_ cells were grown in cultured medium with 2% horse serum for seven days and the transforming growth factor beta-1 (TGF-β1) and collagen I expression were analysed through immunofluorescence staining and western blotting. Results showed that low doses of UT increased both TGB-β1 (Fig. 6a) and collagen I expression (Fig. 6b,c).

In the same way, adipogenic differentiation was evaluated by measuring the mRNA expression of adipogenic markers peroxisome proliferator-activated receptor gamma (PPAR-γ) and fatty acid-binding protein 4 (FABP4) using real-time RT-PCR. The results showed an increase in both markers 48 h after UT treatment (Fig. 6d,e).

These results suggest that low doses of UT change the differentiation program of myoblasts, inhibiting myogenic differentiation and leading to fibrogenic and adipogenic differentiation. Additional experiments were performed with a mixture of IS (25 µg/mL) and an equivalent dose of PCS (22.6 µg/mL) instead of PC, to analyse whether the mixtures IS plus PC and IS plus PCS have similar effects on myoblast differentiation. Results presented in the Supplementary Figure S2 show that the mixture of IS plus PCS impaired myogenic differentiation by reducing the expression of MHC and enhanced fibrogenic and adipogenic differentiation by increasing the expression of Col I, PPAR-γ and FAB4, in the same way as IS plus PC.

### Uraemic rats showed high muscular fibrosis and adipogenic markers in gastrocnemius muscles

**Figure 7:**
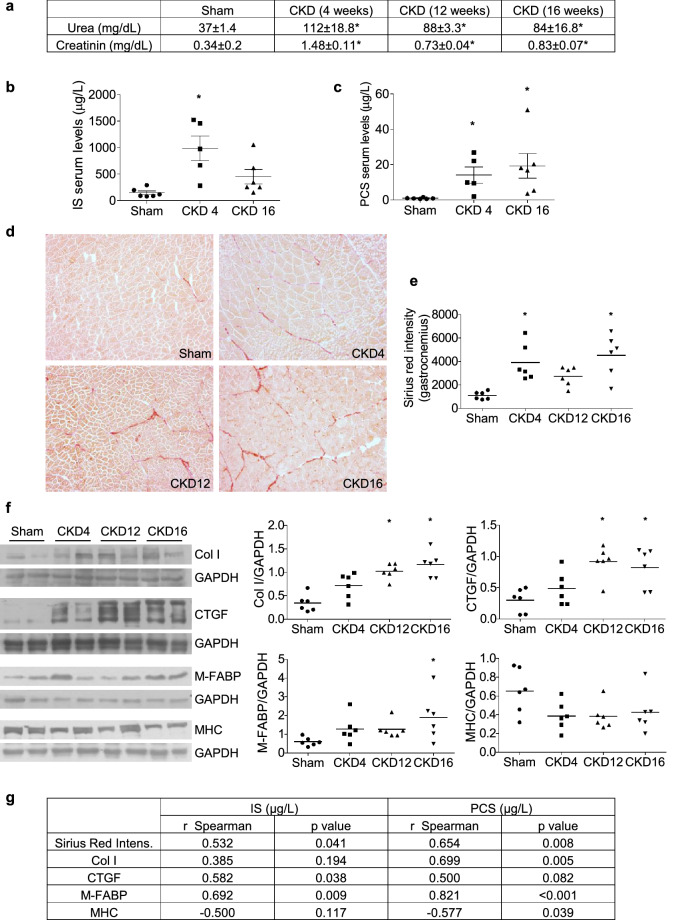
Uraemic rats show a higher rate of fibrosis and adiposis in gastrocnemius muscles. Seven/eight nephrectomised (CKD) or sham operated (Sham) rats were euthanised 4, 12, or 16 weeks after surgery, and gastrocnemii were isolated. (**a**) Urea and creatinine serum concentrations. Results are expressed as mean ± SEM from six animals per group. *p < 0.05 versus Sham. (**b**) Serum concentration of indoxyl sulphate (IS). *p < 0.05 versus Sham. (**c**) Serum concentration of p-cresyl sulphate (PCS). *p < 0.05 versus Sham. (**d**), (**e**) Sirius red staining of gastrocnemii is shown in the left panel. Graph represents the Sirius red intensity measured in six animals from each group by three different researchers. *p < 0.05 versus Sham. (**f**) Collagen I (Col I) and CTGF, Muscle FABP (M-FABP) and Myosin heavy chain (MHC) expression were analysed by western blotting. Typical blots are shown. Full-length blots are presented in Supplementary Figure S3. Graphs show the densitometric analysis of the bands from six animals from each group. *p < 0.05 versus Sham. (**g**) Spearman correlation coefficients, along with p-values, between serum concentrations of IS and PCS with fibrosis markers (Sirius red intensity, Col-I, CTGF), with an adiposis marker (M-FABP) and with a mature myotube marker (MHC).

Next, we analysed whether some of the mechanisms induced by uraemic toxins on cultured myoblast are present in an animal model of CKD. For that, the gastrocnemius muscles in rats with CKD after 7/8 nephrectomy were analysed. Urea and creatinine plasma concentrations increased four weeks after nephrectomy (Fig. 7a). Serum concentration of IS and PCS were significantly elevated in rats with CKD with respect to sham rats (Fig. 7b,c). Rats were euthanised at 4, 12, or 16 weeks after the nephrectomy and gastrocnemius muscles were isolated. Fibrosis was analysed using Sirius red staining. We found that the amount of collagen present in muscle increased with progressing CKD (Fig. 7d,e). The collagen staining was significantly correlated with the serum urea concentration (r = 0.5496, *p* = 0.0054), with the serum creatinine concentration (r = 0.6918, *p* = 0.0002) and with the serum concentration of IS and PCS (Fig. 7g). Collagen I and connective tissue growth factor (CTGF) expressions were also analysed by western blotting, and a significant increase in both was found in uraemic rats (Fig. 7f). Gastrocnemius Col I expression was positively correlated with PCS and CTGF expression was positively correlated with IS serum concentration (Fig. 7g). Additionally, the adipogenic marker muscle and heart fatty acid-binding protein (M-FABP) was analysed in the gastrocnemii by western blotting, and a significant increase was observed in the uraemic rats (Fig. 7f). M-FABP expression was positively correlated with IS and PSC serum concentration (Fig. 7g). Finally, diminished expression of MHC was detected in the muscles of uraemic rats (Fig. 7f), which was negatively correlated with serum PCS levels (Fig. 7g).

## Discussion

Sarcopenia is a condition that is commonly found in patients with CKD. A primary mechanism regulating the loss of muscular mass and strength, associated with sarcopenia, is the disturbance of the muscular regeneration process. This process, triggered in an adult muscle after injury or by inflammatory mediators^23^, is a set of well-coordinated events that starts with the activation of satellite cells, the proliferation of myoblasts, and their myogenic differentiation. Finally, differentiated myoblasts fuse to themselves to form new myotubes. Recently, the role of UT in sarcopenia has been described^18–22^. We present in this work a mechanistic study about the UT effects on the skeletal muscular regeneration process. Results obtained using cultured myoblasts demonstrate that UT partially avoid the normal development of events occurring in this process, suggesting that they could play a role in sarcopenia, even al low concentration.

In this study, a combination of two UT, IS and PC (acting as a surrogate of p-cresyl sulphate, PCS), which are made by the gut microbiota and accumulate in the organs of patients with CKD, were selected, since they are difficult to remove through haemodialysis. IS and PC contribute to aggravated renal dysfunction^24^ and are directly related to increased mortality rates^25^, cardiovascular disease^26^, and decreased bone mass^27^. The serum concentration of UT increases as the rate of glomerular filtration decreases in patients with CKD^13,28^. Therefore, the effects of UT at two different concentrations were analysed to resemble two different stages of CKD progression. The concentrations of UT used in this work are according to the range of those reported in CKD patients. In this sense, Eloot et al. analysed the concentrations of IS and PC, among other UT, in patients with CKD between stages two and five, and identified a range of 15–92 µg/mL for IS and 59–291 µg/mL for PC^28^. Another study from the European Uremic Toxin Work Group reported that total serum levels of IS and PC in the haemodialysis population reached 236 µg/mL for IS and 105 µg/mL for PC^13^. In this study, low doses of UT, 25 µg/mL IS and 10 µg/mL PC, which are similar to those found in the serum of patients in the initial stages of CKD, and high doses of UT, 100 µg/mL of each, were used to mimic advanced stages of CKD. These doses are similar to those used by other authors in cellular models^29,30^. The effect on the muscular regeneration process was evaluated at two levels: first, on the proliferative capacity of myoblasts, and second, on myogenic differentiation.

It was observed that high doses of UT inhibited the proliferative capacity of myoblasts. In fact, after 24 h of treatment, a high percentage of cells remained between phases S and G2, without entering phase M and completing cellular division. Consequently, these cells died through apoptosis. It was determined that treated cells did not progress to cell division by using the fluorescent probe carboxyfluorescein diacetate succinimidyl ester (CFSE), which is an intracellular protein binding dye. Covalently bound CFSE is divided during mitosis equally between daughter cells, discriminating the successive rounds of cell division^31^. In this way, cells treated with UT showed higher fluorescence intensity than control cells. Entry into the mitosis phase is under the control of B-type cyclins, coupled with cdc2^32,33^. When exploring the mechanism involved, no changes in cyclin B, which usually increases in the interphase and reaches a critical threshold just before entry to phase M, were identified. However, a significant increase in cdc2 phosphorylation in Tyr15 was found in the presence of UT. Cdc2 activity is modulated by multiple phosphorylation and dephosphorylation rounds and modifications of its subcellular localization^33^. Since the cdc2-cyclin B complex remained inactive throughout phosphorylation at the Thr14 and Tyr15 residues of cdc2 by Myt1 and Wee1^34,35^, the results of our study indicate that UT inactivate the complex cdc2-Cyclin B, avoiding cell cycle progression. The mechanism involved in cdc2 phosphorylation was not investigated in this study but will be the aim for further experiments.

IS and PC enter cells through the organic anion transporters (OAT)^36,37^. In this study, the OAT inhibitor probenecid was used^38^ to demonstrate that the observed effect was specifically dependent on the UT uptake by muscular cells. It was found that probenecid prevented cell cycle arrest and apoptosis in the presence of high doses of UT, indicating that the effects found were specific to the UT.

Finally, whether high doses of UT promote senescence or apoptosis in myoblasts was analysed. Results indicated that UT did not induce cellular senescence but did induce a significant increase in apoptosis. The link between some UT and apoptosis has previously been described in vascular and renal cells^39–41^, but never before in skeletal myoblasts.

To investigate what happens in skeletal muscles when the UT serum concentration is low, as occurs in the initial stages of CKD, skeletal myoblasts were treated with low doses of UT. It was observed that they did not modify the proliferation capacity of myoblasts and did not induce cell senescence or apoptosis. It has been previously reported that UT induce vascular cell senescence^42^. However, we excluded this possibility in our experimental conditions, even when myoblasts were treated with a high concentration of UT. On the other hand, UT impair myogenic differentiation by reducing the expression of MyoG and decreasing the number of myotubes formed. In contrast, UT increase TGF-β1 and collagen I expression as well as the expression of adipogenic markers FABP4 and PPAR-γ. Our data suggest that in the presence of low doses of UT, myoblasts change the myogenic differentiation program leading to a fibro-adipogenic phenotype. This change in cell fate can be the cause of the appearance of fibrosis and fat deposition into skeletal muscles^43,44^. During sarcopenia, both phenomena occur and have been directly related to loss in force generation^45^.

However, the relevance of the results obtained in vitro must be evaluated considering that we have used p-cresol instead of p-cresyl sulfate, which is the majority p-cresol derivate present in the plasma of the CKD patient. There is not enough evidence in the literature reporting that the effects of both compounds are very different. In this sense, several studies lead us to consider that in vitro effects could be rather similar. Both PC and PCS induce cell migration in TSGH8301 cancer cells^46,47^, induce reactive oxygen species (ROS) production and cytotoxicity in endothelial cells^29,48^, and in tubular cells, PC and PCS induce autophagy and apoptosis processes^49,50^. Consistent with the above mentioned studies, we have demonstrated that the substitution of PC by an equivalent quantity of PCS in some of the experiments performed, does not change the results obtained. However, we cannot ensure that the observed effects in our in vitro model would occur in the same way in CKD patients.

According to the results of this study, a role for IS in uraemic sarcopenia has previously been described. IS induces myotube atrophy^18^, impairs myogenic differentiation^19^, and produces metabolic changes^20^ and mitochondrial dysfunction in C_2_C_12_ cells^22^. The present study demonstrates that low doses of UT not only impair the myogenic program but also change the myoblast cellular fate, leading to the expression of TGF-β1, collagen I, and adipogenic markers. The relationship between UT and cardiac and renal fibrosis has been extensively studied^51^. IS increases TGF-β1 expression and collagen I in renal cells and cardiac fibroblasts^52,53^. PC, as well as PCS, induces fibrosis in kidney tubular cells and cardiomyocytes^54,55^, and promotes the redistribution of fat in the body, thereby modifying insulin resistance^56^. However, the role of UT in skeletal muscle fibrosis and fat deposition related to sarcopenia has never been analysed previously.

Furthermore, the results obtained in the nephrectomised rats confirmed a relationship between uraemia and muscular fibrosis and adiposis. Serum urea and creatinine concentration were used as a symptom of uraemia and chronic kidney disease^57,58^, and we found that the serum concentration of IS and PCS was higher in all rats with CKD used in this study. In our experiments, gastrocnemius muscles isolated from uraemic rats showed increased expression of CTGF and collagen I and fibrosis linked to increased serum urea and creatine concentration, suggesting that muscular fibrosis was linked to CKD. Moreover, the increase in fibrosis and adiposis markers positively correlated with the serum concentration of uraemic toxins, indicating that those rats with higher concentration of serum uraemic toxins also had higher fibrosis and adiposis markers and lower expression of MHC.

In summary, this mechanistic study presents the effect of UT on the muscular regeneration process. The study demonstrates that this effect differs depending on the UT concentration. Low doses of UT, replicating the early stages of CKD, would impair the myogenic program, changing the myoblast cell fate, reducing the number of myotubes formed, and priming the fibrogenic and adipogenic differentiation of the myoblasts. This could be a mechanism involved in the appearance of adipose tissue and fibrosis in skeletal muscle. In contrast, high doses of UT, like those found in the late stages of CKD, would induce a dramatic arrest in the cell cycle of myoblasts, interrupting their proliferation and leading to cell apoptosis. Moreover, we observed that the UT increment in the serum of rats with CKD is significantly correlated to markers of muscular fibrosis and adiposis. The clinical significance of these mechanisms in CKD patients needs further experiments.

## Materials and methods

### Cell culture

Mouse myoblast cell line, C_2_C_12_, was purchased from the American Type Culture Collection (Manassas, VA, USA). Cells were cultured in Dulbecco’s Modified Eagle Media (DMEM) with 4.5 g/L glucose, 10% FBS, 100 μg/mL streptomycin and 100 U/mL penicillin. Cells grew in a 95% air and 5% CO_2_ atmosphere. Cells were used at passages 3–10.

### Uraemic toxins

IS was dissolved in water 12.5 mg/mL and PC was dissolved in methanol 100 mg/mL as previously described^29^. PCS was dissolved in water 12.5 mg/mL. All of them were later diluted, to reach the final concentration, in DMEM containing 10% FBS or 2% horse serum (HS), before adding to cell culture. Water and methanol as vehicle were added to control cells under the same conditions as the treated cells.

### Experimental design

For cell proliferation assays, cells were grown with 0% or 10% FBS culture media with or without UT (IS and PC). For differentiation assays, cells were grown in culture medium with 2% HS for seven days to allow myogenic differentiation with or without UT. Low doses (25 μg/mL IS and 10 μg/mL PC) resembled the UT serum concentration found in the early stages of CKD. High doses (100 μg/mL of each) corresponded to the UT serum concentration found in the advanced stages of CKD. Selected experiments were performed using PCS instead of PC at the same molarity of PC (22.6 μg/mL for low doses or 226 μg/mL for high doses).

### Animal studies

Twelve-week-old Wistar rats, housed in a pathogen-free temperature-controlled room (22 ± 2 °C), were used for the experiments. Chronic renal failure was induced in Wistar rats by performing a seven/eight nephrectomy. The control group were sham-operated rats (Sham). Following surgery, rats were housed in wire cages and provided a standard diet and water ad libitum for 4, 12, or 16 weeks. At the time of euthanasia, rats were anaesthetised, and gastrocnemius muscle samples were obtained and conserved either in RNA later solution for protein and RNA extraction or conserved at − 80 °C for histological analysis. Serum urea and creatinine levels were measured using a multichannel auto analyser (Hitachi).

All the experimental protocols were made according to the European Union regulations (EU Directive 2010/63/EU and to the Guide for the Care and Use of Laboratory Animals published by the US National Institute of Health (NIH Publication No.85-23, revised 1996). The study was revised and approved by the Principado de Asturias Government (PROAE 15/2015).

### Protein extraction and immunoblot analysis

Proteins from gastrocnemius muscles from rats and from treated cells were isolated using a lysis buffer containing 20 mM Tris–HCl pH 7.5, 150 mM NaCl, 1 mM EDTA, 1 mM EGTA, 1% Triton X-100, 0.1% sodium deoxycholate, 10 mM sodium pyrophosphate buffer with a protease inhibitor cocktail. Lysates were centrifuged at 13,000 rpm for 30 min at 4 °C. A BioRad protein assay kit was used to quantify protein concentration. Protein samples were run onto 8–12% SDS–polyacrylamide gels (PAGE) under reducing conditions followed by transference onto PVDF membranes in all cases except for collagen I which need non-reducing conditions. Membranes were blocked with 5% non-fat dry milk dissolved in Tween Tris buffered saline (TTBS) (2 mM Tris–HCl, pH 7.5, 15 mM NaCl, 0.05% Tween-20) for 1 h at room temperature (R/T), and then incubated with the primary antibodies (PCNA 1:1000, MHC 1:2000, MyoG 1:500, Collagen I 1:2000, Cyclin B1 1:1000, cdc2 1:1000, p-cdc2 1:1000, CTGF 1:1500 or M-FABP 1:1000) O/N at 4ºC; followed by incubation with secondary antibodies 1 h at R/T. The bands were visualised using a chemiluminescence reagent detection system and analysed by densitometry with ImageJ software 2.6 (http://rsbweb.nih.gov/ij/). Blots were then reblotted with a house-keeping gene, mouse anti- Glyceraldehyde 3-phosphate dehydrogenase (GAPDH) antibody or Actin antibody, to normalise the protein levels.

### Quantitative RT-PCR

Total RNA was isolated from C_2_C_12_ cells using TRIzol reagent according to the manufacturer’s protocol. cDNA was synthesised using a High-Capacity cDNA Reverse Transcription Kit. Gene expression was measured through quantitative PCR (ABI Prism 7500 Fast Real-Time PCR System) and analysed using 7500 Fast Sequence Detection Software v.1.3.1 (Applied Biosystems Inc., Foster City, CA, USA) using specific mouse TaqMan genes and Double delta Ct method. TaqMan genes: FABP4 (Mm00445878_m1), PPAR-γ (Mm00440940_m1), and the endogenous control GAPDH (Mm99999915_g1).

### Measurement SA-β-gal activity

Myoblasts were cultured on microscope cover slides. After 24 h of treatment with serum-free DMEM, they were incubated with or without IS and PC for 24, 48 and 72 h in serum-free DMEM. Cellular senescence was measured by SA-β-gal activity using fluorescence confocal microscopy, with the substrate C_12_FDG (33 μM) added to cultured cells for 2 h after UT treatment. Then, cells were washed twice with phosphate buffered saline (PBS), and fixed with 4% paraformaldehyde for 15 min. Subsequently, cells were rewashed and mounted in ProLong Gold antifade reagent with DAPI. SA-Green immunofluorescence was measured using a LEICA TCS-SP5 confocal microscope (Leica Microsystems; GmbH, Mannheim, Germany) with a 488 nm argon laser. An argon laser at 405 nm was used to detect DAPI. Images were obtained, and fluorescence intensity was analysed using ImageJ software (http://rsbweb.nih.gov/ij/).

### In vitro wound-healing model

Cells were serum starved for 24 h and then treated with 10% FBS culture medium containing IS and PC or vehicle for 24 and 48 h. Afterwards, the treatment was removed, and the monolayer was scratched using a needle to create a 0.6 mm-wide wound. Cells were stimulated to proliferate with standard culture medium supplemented with 10% FBS for 24 h. Images of the wound areas were taken with a Moticam microscope (Motics Microscopes) 0, 18, and 24 h post-wounding. The scratch wound area was quantified using ImageJ software (http://rsbweb.nih.gov/ij/).

### Immunocytochemistry

Cells grew on microscope cover slides with 2% HS to allow myogenic differentiation with or without UT for seven days. We used the expression of MHC, MyoG, and desmin to detect myotube formation by immunofluorescence using a confocal microscope. In contrast, we analysed the expression of collagen I, TGF-β1, and α-Smooth muscle actin (α-SMA) to determine fibrogenic differentiation.

After UT treatment, cells were washed twice with PBS and fixed with 4% paraformaldehyde (15 min at R/T), then permeabilised with 0.5% Triton X-100 for 10 min at R/T. Later, cells were blocked with 5% BSA (60 min at R/T), and incubated with either mouse anti-myogenin (1:100), rabbit anti-desmin (1:500), rabbit anti-collagen I (1:200), rabbit anti-TGF-β1 (1:100) or mouse anti-α-SMA (1:200) overnight at 4 °C or with mouse anti-MHC (1:100) for 2 h at R/T.

Next, cells were washed in PBS and incubated for 1 h with a mix of 200-fold diluted goat anti-rabbit IgG labelled with Alexa Fluor 488 to detect rabbit antibodies (green), and 200-fold diluted goat anti-mouse IgG labelled with Alexa Fluor 647 to detect mouse antibodies (red). Cover slides were mounted on ProLong Gold antifade reagent with DAPI overnight. Preparations were analysed using confocal microscope LEICA TCS-SP5. Pictures were taken and ImageJ software was used to measure fluorescence intensity (http://rsbweb.nih.gov/ij/).

### Analysis of Chromosomal Condensation

For proliferation assays, cells were grown on microscope cover slides. After 24 h of starvation, C_2_C_12_ were cultured in 10% FBS DMEM with or without UT for 24 h. To induce cell cycle arrest at mitosis, colcemid (0.1 μg/mL) was added at the same time as the UT. After treatments, cells on the microscope coverslips were analysed as described above but using rabbit anti-acetyl-histone H3 (1:100) and mouse anti-tubulin (1:100) antibodies.

### Cell cycle analysis by DNA content

After UT treatment, cells were trypsinised, centrifuged at 1300 rpm for 6 min, and resuspended in 500 µL of cold PBS. Next, cells were fixed in 2 mL ice-cold 70% ethanol (30 min at − 20 °C), and following cell fixation, samples were centrifuged (1300 rpm for 6 min) and resuspended in 500 µL of PBS-RNase (50 µg/mL). Finally, cells were incubated at 37 °C for 30 min, stained with propidium iodide (50 µg/mL) and then analysed at 488 nm on a FACScan flow cytometer (Becton–Dickinson). Results were analysed using WinMDI 2.8 software (developed by Joe Trotter, TSRI, La Jolla, CA, USA) and Cylchred 1.0.2 software (Cardiff University, Wales, UK). Selected experiments were performed by treating myoblasts for 30 min with the organic anion transporter (OAT) inhibitor, probenecid (0.25 mM) before adding the UT.

### Annexin V staining

To analyse apoptosis, FITC Annexin V Apoptosis Detection Kit I (BD Biosciences Europe, Madrid, Spain) was used according to the manufacturer's protocol. After treatments, cells were trypsinised, centrifuged (1300 rpm for 6 min), washed twice with cold PBS and resuspended in 100 µL of 1 × Binding Buffer. 5 μL of FITC Annexin V and 5 μL of propidium iodide were added to the samples, and then they were incubated in the dark (15 min at R/T). Finally, 400 µL of 1 × Binding Buffer was added, and samples were analysed on a FACScan flow cytometer (Becton–Dickinson, Franklin Lakes, NJ, USA). Results were analysed using Cyflogic software (Cyflogic software, CyFlo Ltd, Finland). Selected experiments were performed treating myoblasts for 30 min, before adding the UT, with the OAT inhibitor probenecid (0.25 mM).

### Cell proliferation assay

Cells were stained using a CellTrace CFSE Cell Proliferation Kit (Invitrogen, Thermo Fisher Scientific brand, Madrid, Spain) according to the manufacturer's protocol. Cells grew to the desired density on culture plates and were incubated for 10 min at 37 °C in CellTrace CFSE solution (5 μM). After washing twice with culture media, cells were incubated for 30 min with culture media before analysis, allowing the reagent to attach to cytoplasmic components of cells. The unstimulated parent generation (time 0 h) was analysed on a FACScan flow cytometer (Becton–Dickinson) to establish the maximum of fluorescence intensity. Next, cells were stimulated with UT for 24 and 48 h and the analysis of the fluorescence of the experimental conditions were conducted.

### Sirius red staining

The connective tissues of the gastrocnemius muscle were stained using a Picro-Sirius Red Stain Kit (Abcam, Cambridge, UK). Tissue sections were hydrated in distilled water (5 min) and incubated with Picro-Sirius Red Solution for 20 min. Next, they were rinsed twice each with acetic acid solution and absolute alcohol. Finally, tissues were mounted in DPX mounting medium fast to be observed with a microscope. Images were obtained under 20× magnification and Sirius red intensity was quantified by Image Pro Plus software (http://www.mediacy.com/imageproplus).

### IS and PCS quantification by liquid chromatography–mass spectrometry (LC–MS)

Serum samples from uraemic rats were prepared for free IS and PCS analysis as previously described^59^. Protein precipitation was carried out by adding 200 µL of acetonitrille to a 100 µL aliquot of serum samples, vortex-mixing for 3 min and centrifuging at 20,000 g for 5 min. Then, the supernatant was equally mixed 1:1 (v/v) with 10 mM ammonium acetate buffer for analysis. LC–MS analysis was conducted on a Schimazdu LC/MS-8030 equipped with a triple quadrupole mass analyser (QqQ) and an electrospray source operating in negative ionization mode. Multiple Reaction Monitoring (MRM) mode was used to quantify the extracted metabolites. Standards of each metabolite were prepared in 10 mM ammonium acetate: acetonitrile 1:1 (v/v) for external calibration. For identification of uraemic toxins, liquid chromatography was performed with a C18- Phenomenex Gemini column (5u 110 A 150 × 2 mm). Mobile phase A consisted of 5 mM ammonium acetate and mobile phase B was exclusively composed by pure acetonitrile. The binary gradient used to perform the chromatographic separation is shown in Table 1. The flow rate was maintained at 0.40 mL/min. 10 µL of either the standards mixture or the samples were analysed. Full-scan MS spectra and MS/MS spectra were acquired in order to obtain the maximum number of available transitions for each analyte. N_2_ was used as both nebulizing and drying gas, working at a flow rate of 1.5 and 15.0 mL/min, respectively. The ionization voltage was set at 4.5 kV and the desolvation line (DL) temperature at 250 °C. Collision-induced dissociation was performed using Ar as the collision gas at a pressure of 230 kPa in the collision cell. The detector voltage was set at 1.8 kV and detection was carried out in MRM mode with a dwell time of 100 ms, by monitoring two unique transitions for each compound. The MRM transitions were selected based on the higher intensities observed for the different ion fragments. The most sensitive transition was selected for quantification (Table 2) whereas the others were used for confirmation purposes.

**Table 1:** Gradient mode.

Time (min)	Phase A (%)	Phase B (%)
0	95	5
7	0	100
8	0	100
8.5	95	5
10	95	5

**Table 2:** MS/MS transitions.

**PCS**	
Quantifier (m/z): 187.1 < 107.1	CE: 20
Qualifier (m/z): 187.1 > 79.95	CE: 21
**IS**	
Quantifier (m/z): 212.1 > 80.0	CE: 21
Quantifier (m/z): 212.1 < 132.0	CE: 20

### Statistical analysis

Results were calculated as the mean ± standard error of a variable number of independent experiments, as detailed in the figure captions. GRAPHPAD Prism 5 software (GraphPad Prism Software Inc., San Diego, CA, USA) was used for statistical analysis. One-way or two-way ANOVAs followed by Dunnet’s post-tests for experiments compared with control cells or by Bonferroni post-tests for multiple comparisons were used. Experiments performed on animals were analysed using one-way ANOVAs and correlations were analysed using the non-parametric Spearman correlation. Statistical significance was fixed as *p* < 0.05.

## Supplementary information

Supplementary Information 1.

## Data Availability

All data generated or analysed during this study are included in this published article (and its Supplementary Information files).
