# Supplementary material for: Uraemic toxins impair skeletal muscle regeneration by inhibiting myoblast proliferation, reducing myogenic differentiation, and promoting muscular fibrosis
Source: Sci Rep. 2021 Jan 12;11:512. doi: 10.1038/s41598-020-79186-1 (PMC7804102; doi:10.1038/s41598-020-79186-1)
Supplement: Supplementary file 1 — Supplementary Information 1. [file 41598_2020_79186_MOESM1_ESM.pdf]

Uraemic toxins impair skeletal muscle regeneration by inhibiting myoblast proliferation, reducing miogenic differentiation, and promoting muscular fibrosis.

Elena Alcalde-Estévez[1], Patricia Sosa[1], Ana Asenjo-Bueno[2], Patricia Plaza[2], Gemma Olmos[1,3,4,*], Manuel Naves[5], Diego Rodríguez-Puyol[3,4,6], Susana López-Ongil[2,3,4,7], María P. Ruiz-Torres[1,3,4,7].

[1]Departamento Biología de Sistemas, Universidad de Alcalá, Alcalá de Henares, Madrid, SPAIN.

[2]Unidad de Investigación de la Fundación para la Investigación Biomédica del Hospital Universitario Príncipe de Asturias, Alcalá de Henares, Madrid, SPAIN.

[3]Instituto Reina Sofía de Investigación Nefrológica, IRSIN, Madrid, SPAIN.

[4]Area 3-Fisiología y Fisiopatología Renal y Vascular del IRYCIS, Madrid, SPAIN.

[5]Unidad de Gestión Clínica de Metabolismo Óseo. Hospital Universitario Central de Asturias. ISPA, Oviedo, SPAIN.

[6]Departamento de Medicina y Especialidades Médicas, Universidad de Alcalá y Servicio de Nefrología del Hospital Universitario Príncipe de Asturias, Alcalá de Henares, Madrid, SPAIN.

[7]These authors contributed equally to the manuscript: Susana López-Ongil and María P. Ruiz-Torres.

All authors are part of Programa Redes REDinREN from the FIS (RD016/0009/0017; RD016/0009/0018), and FRIAT, SPAIN.

[*]Corresponding author*:* Gemma Olmos. Email: gemma.olmos@uah.es

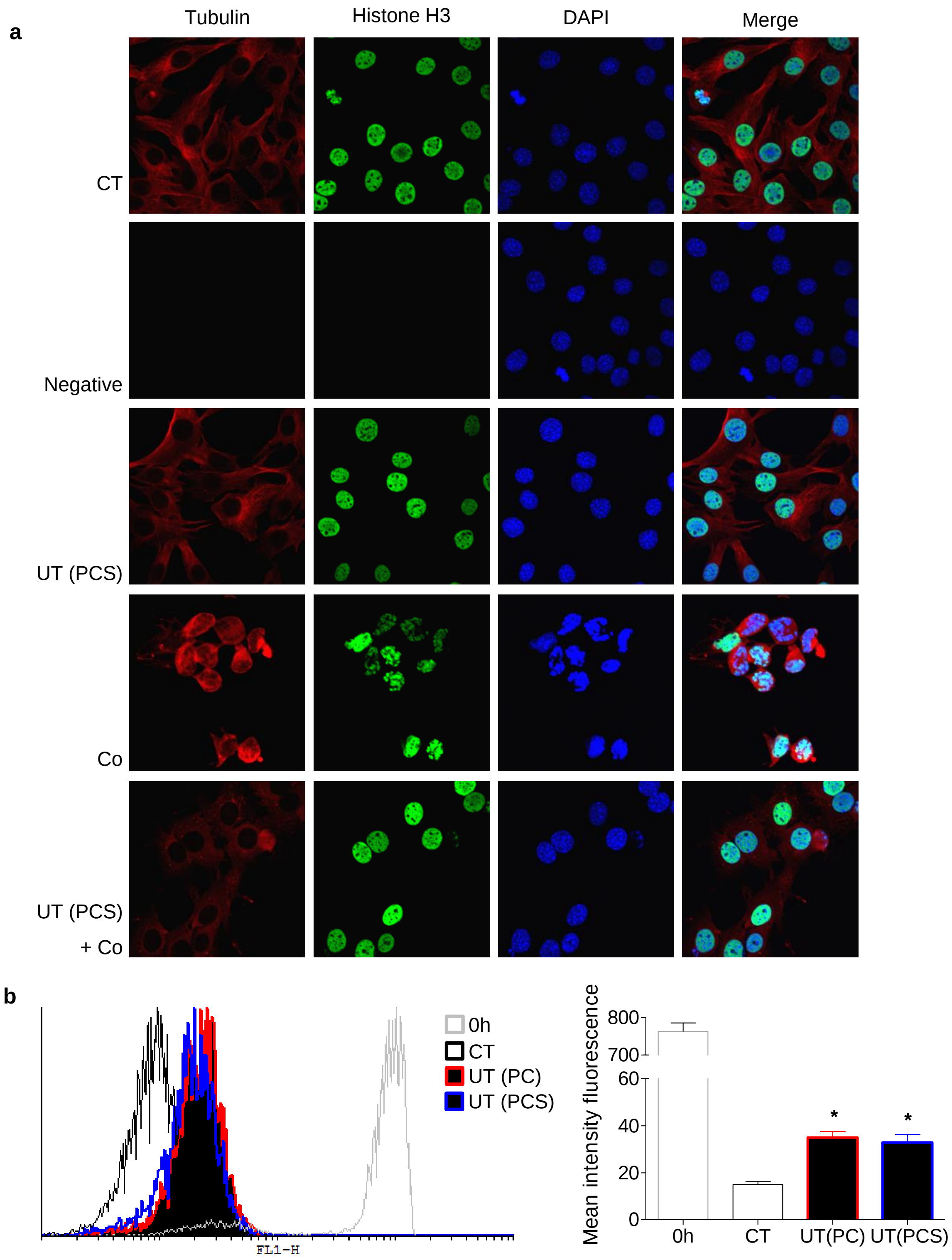

Supplementary Figure S1. High doses of UT (PCS + IS) prevented entry into phase M and reduced the proliferation capacity of cultured murine myoblasts. a C_2C_{12} cells were treated with a mixture of 100 µg/mL IS and 226 µg/mL PCS (UT (PCS)) for 24 hours in the presence or absence of 0.1 mg/mL colcemid (Co). Tubulin (red) and Histone H3 (green) expression and DAPI (blue) were analysed by immunofluorescence by using a confocal microscope. A representative experiment with negative staining control is shown. **b** Cell proliferation was monitored using CFSE solution by flow cytometry during 48 hours. C_2C_{12} cells were treated with a mixture of 100 µg/mL IS and 100 µg/mL PC (UT (PC)) or 100 µg/mL IS and 226 µg/mL PCS (UT (PCS)). A representative experiment is shown. The bar graph represents the mean fluorescence intensity (MFI). The results are mean ± SEM from three experiments. *$p<0.05$ vs CT.

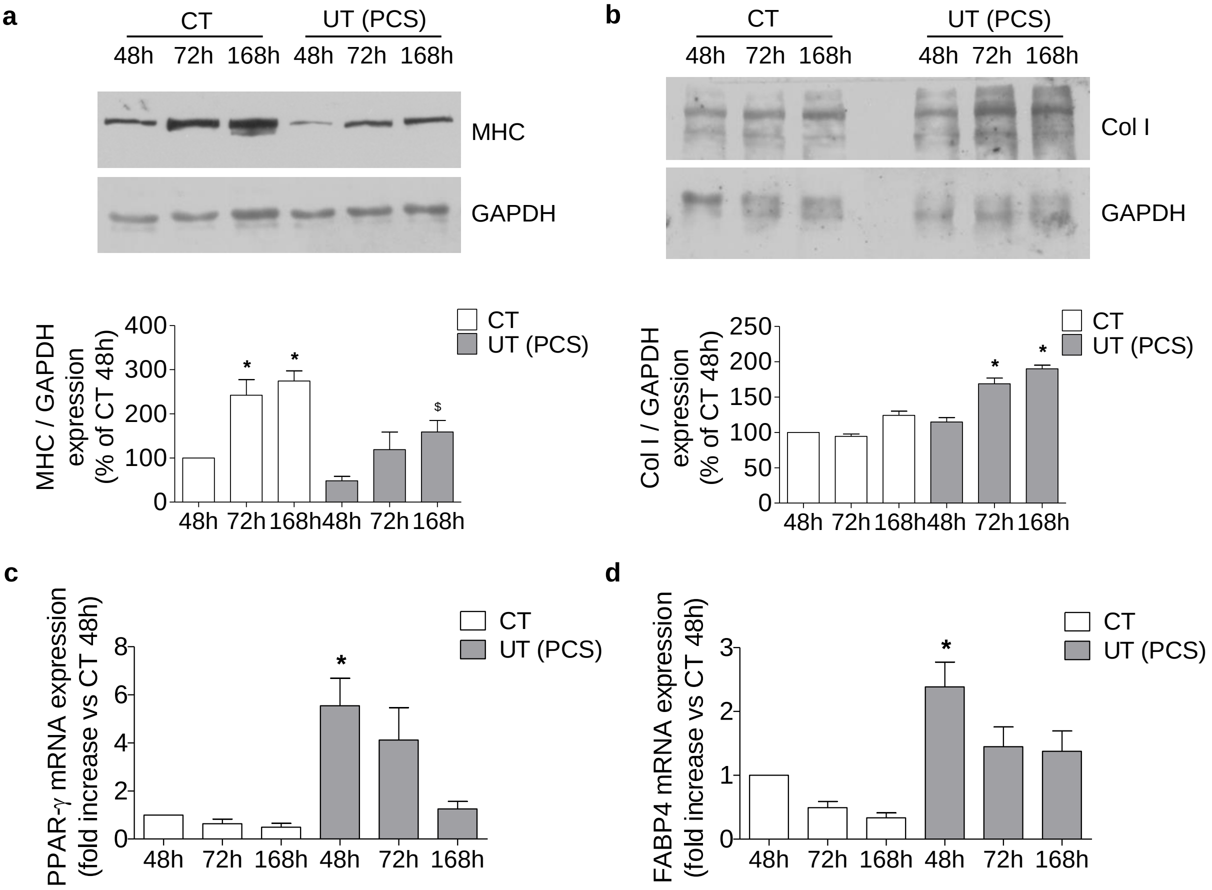

Supplementary Figure S2. Low doses of UT (IS + PCS) impaired the myogenic differentiation and promoted fibrogenic and adipogenic differentiation in murine cultured myoblasts. C_2C_{12} cells grew with 2% horse serum for 48, 72, and 168 hours to allow myogenic differentiation in the presence of a mixture of 25 µg/mL IS and 22.6 µg/mL PCS (UT (PCS)) or vehicle (CT). **a** MHC expression was measured by western blotting. A typical blot is shown. The full-length blot is presented in Supplementary Figure 3. The densitometric analysis of the bands is represented in bar graph. Results are the percentage respect to control 48h and are the mean ± SEM from four experiments. *p<0.05 vs CT 48h. $^{\$}$p<0.05 vs CT 168h. **b** Collagen I (Col I) protein expression was quantified by western blotting. A typical blot is shown. The full-length blot is presented in Supplementary Figure 3. The bar graph shows the densitometric analysis of the bands. Results are percentage of control 48h and are the mean ± SEM from three experiments. *p<0.05 vs CT 48h. **c** PPAR-γ mRNA expression was analysed by RT-PCR. Results are mean ± SEM from four experiments. *p<0.05 vs CT 48h. **d** FABP4 mRNA expression was analysed by RT-PCR. Results are mean ± SEM from three experiments. *p<0.05 vs CT 48h.

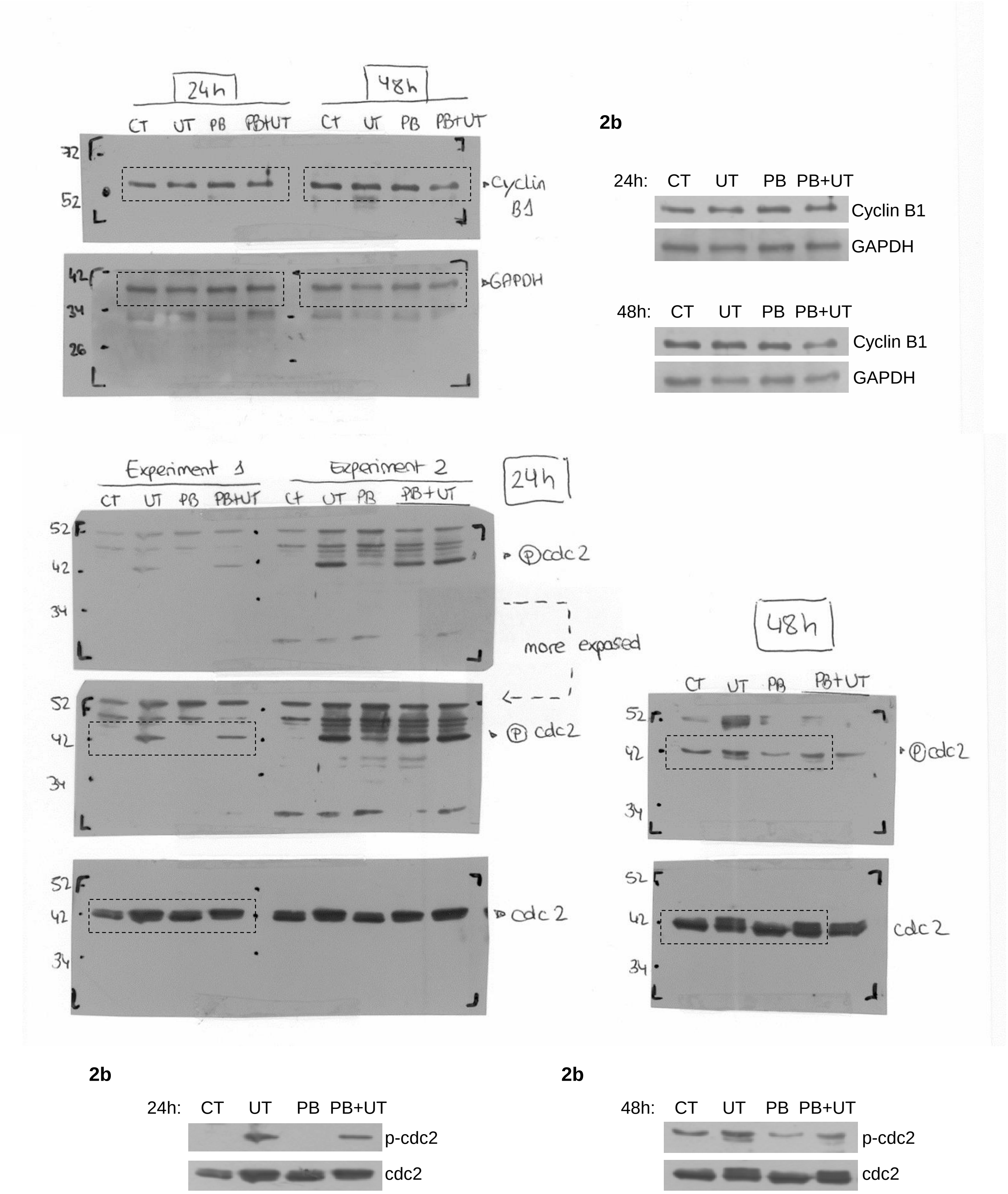

Supplementary Figure S3. Full-length blots included in Figures 2, 4, 5, 6 , 7 and Supplementary S2.

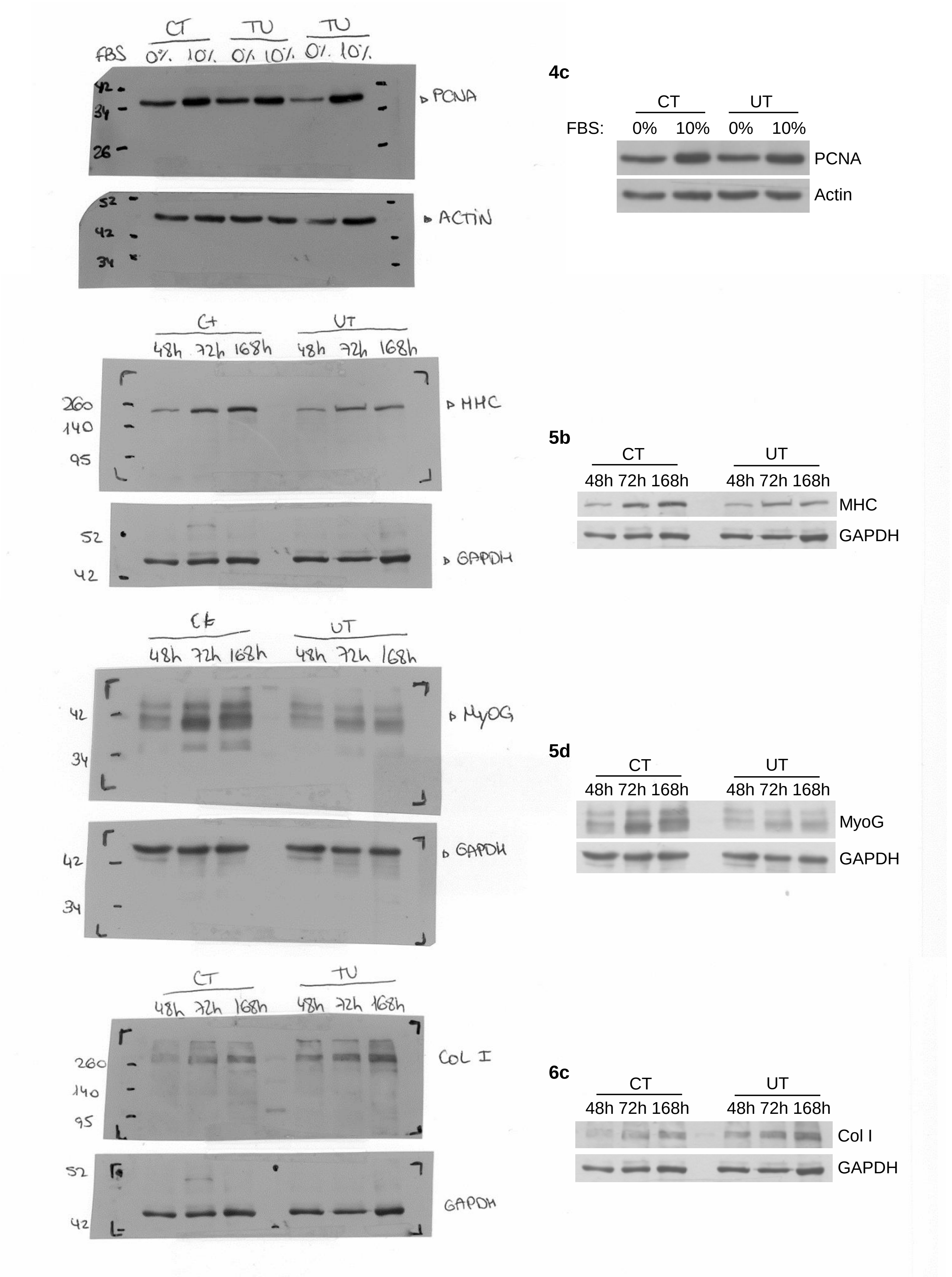

Supplementary Figure S3. Full-length blots included in Figures 2, 4, 5, 6 , 7 and Supplementary S2.

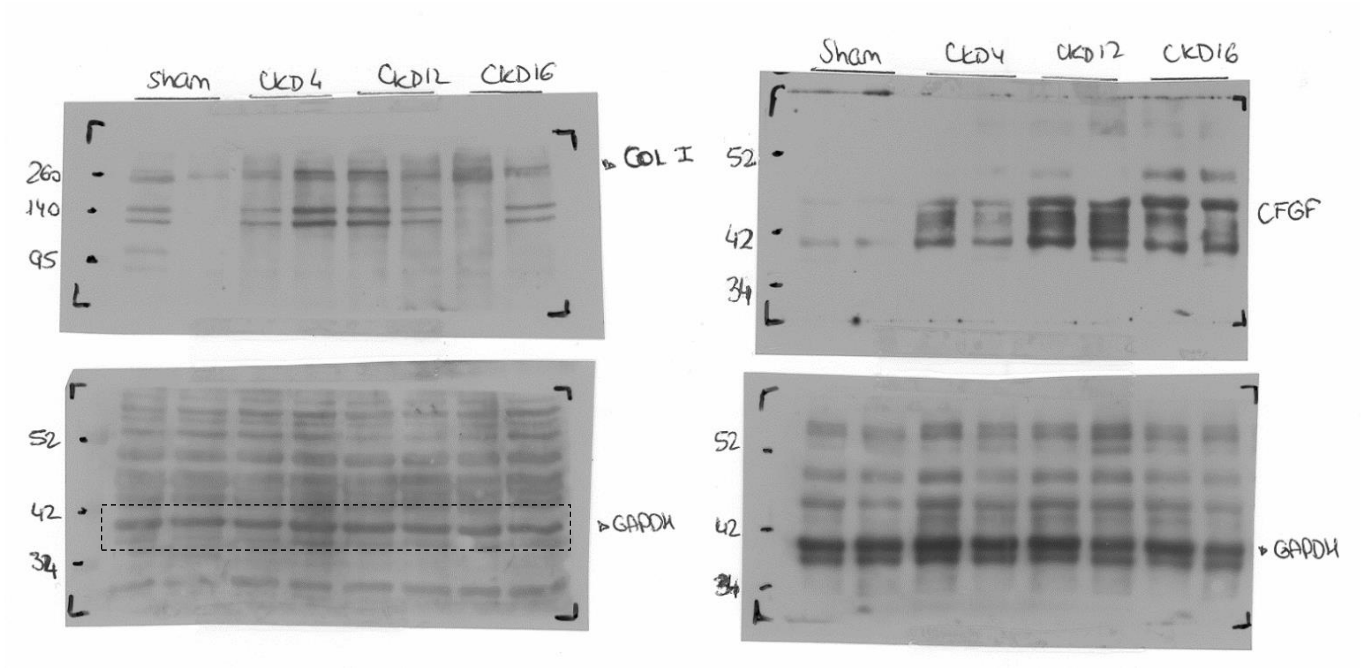

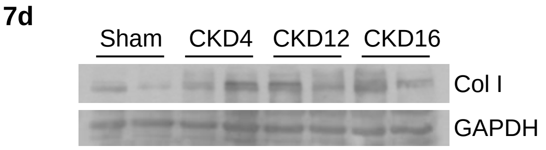

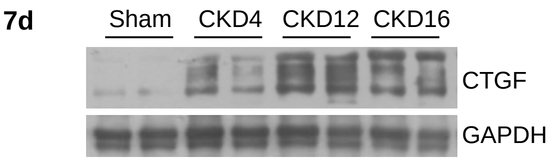

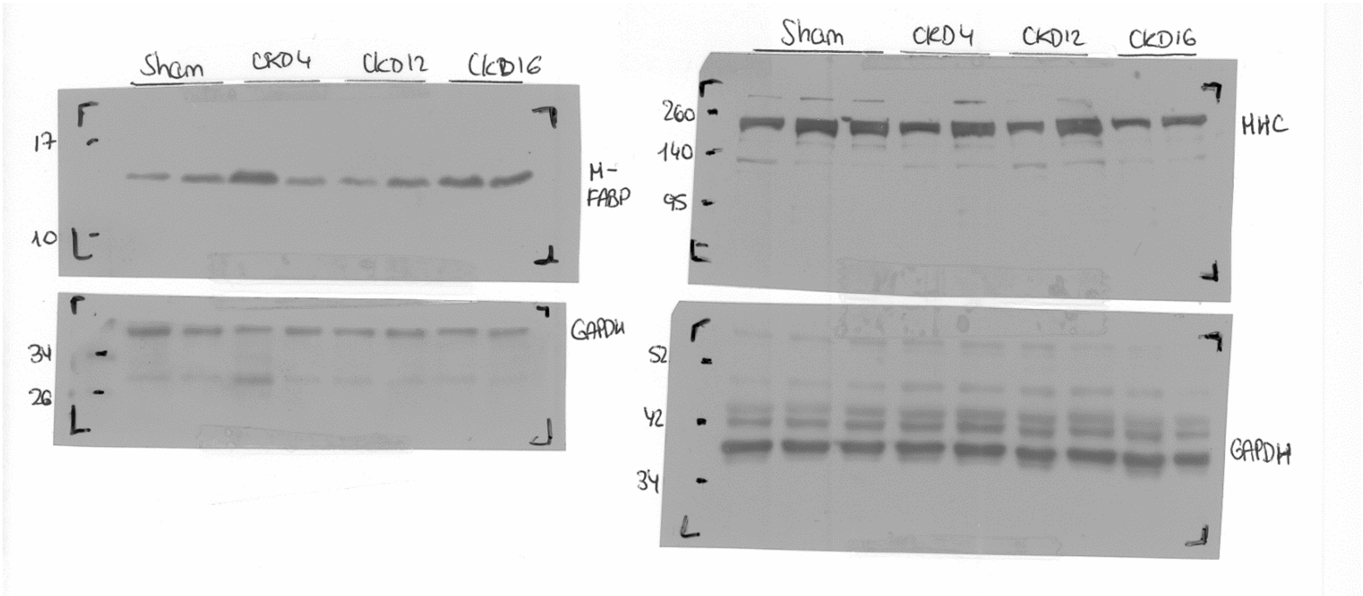

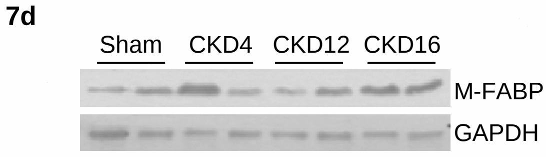

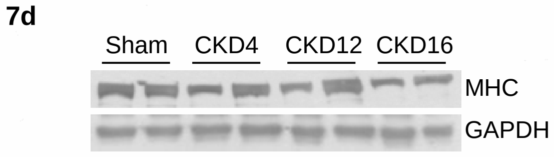

Supplementary Figure S3. Full-length blots included in Figures 2, 4, 5, 6 , 7 and Supplementary S2.

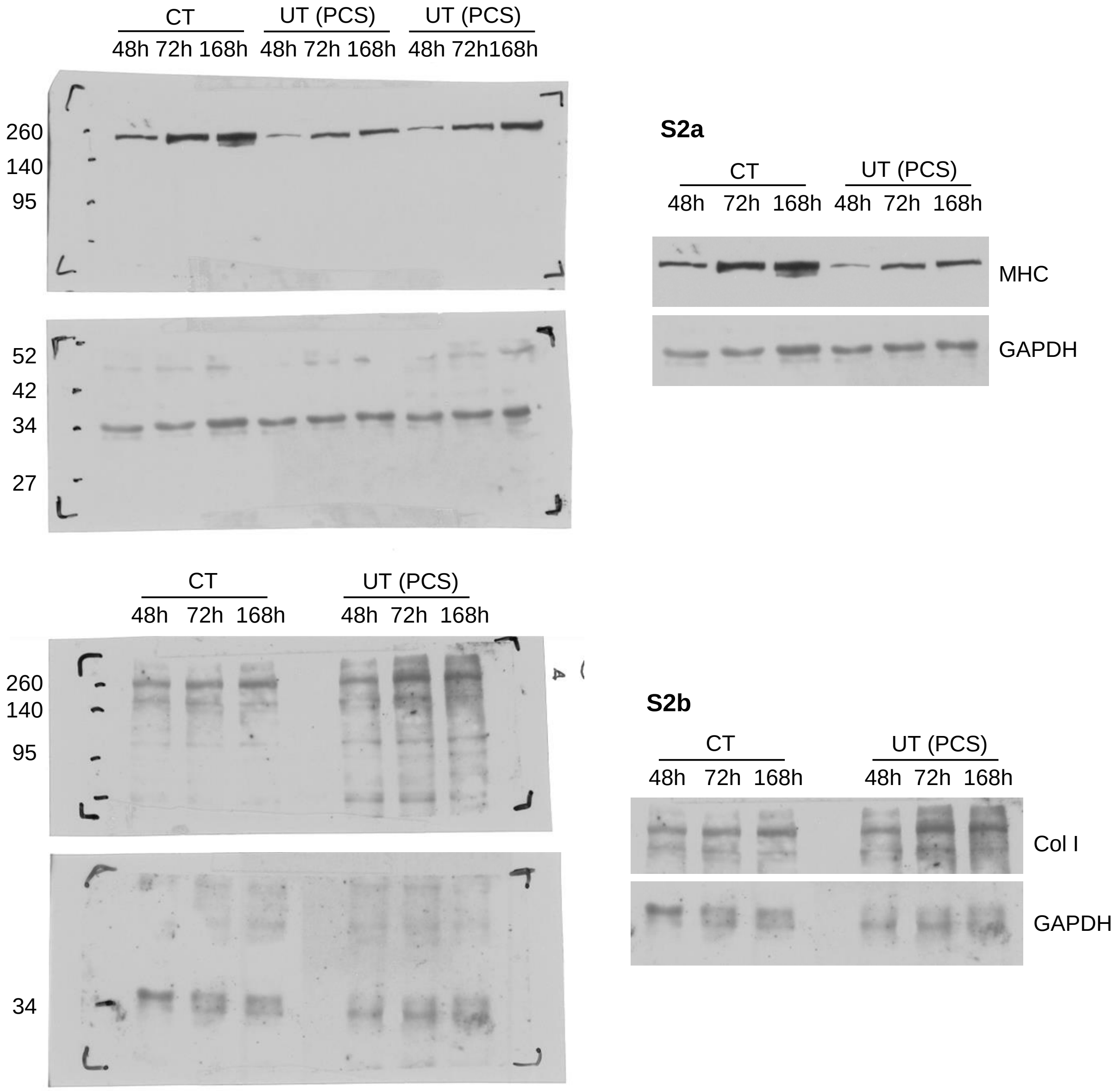

Supplementary Figure S3. Full-length blots included in Figures 2, 4, 5, 6 , 7 and Supplementary S2.

Supplementary information

Materials

Dulbecco´s Modified Eagle´s Medium (DMEM) was obtained from ATCC (Basel, Switzerland). Culture plates, penicillin, streptomycin, BCA protein assay reagent for measuring protein concentration, blueStar-pre-stained protein marker, CL-Xposure films and secondary horseradish peroxidase-conjugated goat anti-mouse IgG were obtained from Cultek (Thermo Fisher Scientific, Madrid, Spain) and the Supersignal West Pico was from Pierce (Thermo Fisher Scientific, Madrid, Spain). ProLong Gold antifade reagent with DAPI and fluorogenic ImaGene green C_{12}FDG (5-dodecanoyl-aminofluorescein di-β-D-galactopyranoside) were purchased from Molecular Probes (Thermo Fisher Scientific, Madrid, Spain). PVDF membranes and electrophoresis equipment were obtained from Bio-Rad Laboratories (Richmond, CA, USA). Acrylamide-bisacrylamide was from Hispanlab-Pronadisa (Madrid, Spain). Protease Inhibitor Cocktail Tablets and FastStart Universal Probe Master were obtained from Roche Diagnostics S.L. (Barcelona, Spain). TRIzol reagent was provided by Ambion-Life Technologies (Thermo Fisher Scientific, Madrid, Spain). The FITC Annexin V apoptosis detection kit was purchased from BD Biosciences Europe (Madrid, Spain). TaqMan gene expression assays from mouse and High-capacity cDNA reverse transcription kit and TaqMan genes were provided by Applied Biosystems (Thermo Fisher Scientific, Madrid, Spain). Colcemid (Demecolcine) Solution was obtained from Biological Industries (Cromwell CT, USA). The secondary horseradish peroxidase-conjugated goat anti-mouse IgG was obtained from Dako (Glostrup, Denmark). Picro-Sirius Red Stain Kit was from Abcam (Cambridge, UK). Cell Proliferation Kit, CellTrace CFSE, Alexa Fluor 647 goat anti-mouse and Alexa Fluor 488 goat anti-rabbit were from Invitrogen (Thermo Fisher Scientific, Madrid, Spain). Probenecid (water soluble) was purchased from Thermo Fisher Scientific, Madrid, Spain. PCNA, MyoG, cyclin B1, cdc2, phospho-cdc2 and M-FABP antibodies were obtained from Santa Cruz BioTechnology (Sta. Cruz, CA, USA). Acetyl-Histone H3 (Lys9) antibody was obtained from Cell Signalling (IZASA, Barcelona, Spain). Desmin, collagen I, TGF-β1, and CTGF antibodies were obtained from Abcam (Cambridge, UK). Anti-MHC antibody was obtained from R&D Systems (Minneapolis, MN, USA). DPX mounting medium fast was provided by PanReac AppliChem (Barcelona, Spain). Potassium p-cresyl sulphate was purchased from Tokyo Chemical Industry (Tokyo, Japan). Tubulin, GAPDH, and α-SMA antibodies, indoxyl sulphate potassium salt, p-cresol, propidium iodide, FBS, horse serum and all other drugs and reagents (unless otherwise indicated) were provided by Sigma-Aldrich-Fluka Chemical Co. (St. Louis, MO, USA).
